# Supplementary material for: In-memory photonic dot-product engine with electrically programmable weight banks
Source: Nat Commun. 2023 May 20;14:2887. doi: 10.1038/s41467-023-38473-x (PMC10199927; doi:10.1038/s41467-023-38473-x)
Supplement: Supplementary file 1 — Supplementary Information [file 41467_2023_38473_MOESM1_ESM.pdf]

Supplementary information

In-memory photonic dot-product engine with electrically programmable weight banks

Wen Zhou,[1,†] Bowei Dong,[1,†] Nikolaos Farmakidis,[1] Xuan Li,[1] Nathan Youngblood,[1,2] Kairan Huang,[1] Yuhan He,[1] C. David Wright,[3] Wolfram H. P. Pernice,[4,5] and Harish Bhaskaran[1,*]

[1]Department of Materials, University of Oxford, Parks Road, OX1 3PH Oxford, United Kingdom
[2]Current Address: Department of Electrical and Computer Engineering, University of Pittsburgh, Pittsburgh, PA, 15261, USA
[3]Department of Engineering, University of Exeter, Exeter EX4 4QF, United Kingdom
[4]Institute of Physics, University of Münster, Heisenbergstr. 11, 48149 Münster, Germany
[5]Heidelberg University Kirchhoff-Institute for Physics Im Neuenheimer Feld 227, 69120 Heidelberg, Germany

[†]These authors contributed equally: W. Zhou and B. Dong
[*]Corresponding author: harish.bhaskaran@materials.ox.ac.uk

Supplementary Note 1: IV scan of a silicon-on-insulator (SOI) microheater

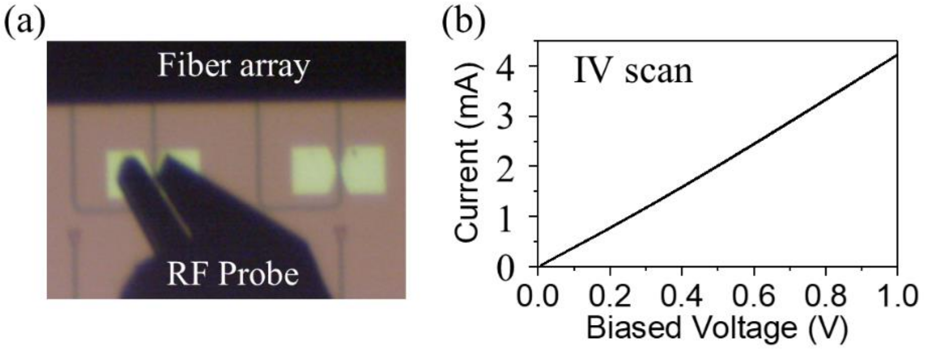

Supplementary Figure 1 IV scan of a heavily P^{++} doped SOI microheater. (a) An experimental setup for characterizing a microheater. **(b)** Net current versus biased voltage between 0 and 1 V.

Supplementary Figure 1a shows a top-view micrograph of our experimental setup for electrical testing of a heavily P^{++} doped silicon-on-insulator (SOI) waveguide microheater. A ground-signal (GS) radiofrequency (RF) probe was used to contact the aluminum (Al) pads. The grating couplers were separated far away from the microheater for fiber array positioning. Supplementary Figure 1b shows IV scan across a doping region with a total resistance of 236.9 Ohm. It exhibits a linear response that reveals the good Ohmic contact between Al pads and doped silicon.

Supplementary Note 2: Optical microscopic images of devices in each fabrication step

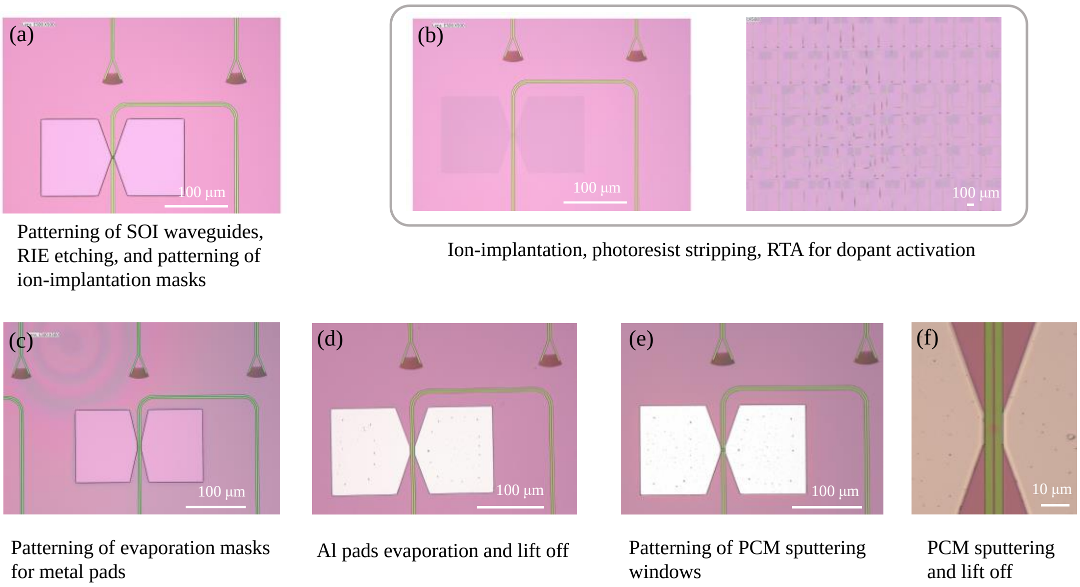

Supplementary Figure 2 Optical microscopic images of devices in each fabrication step. (a) Patterning of SOI waveguides using electron beam lithography (EBL) and reactive ion etching (RIE) etching, and patterning of ion-implantation masks. **(b)** Ion-implantation, photoresist stripping, rapid thermal annealing (RTA) for dopant activation. **(c)** Patterning of thermal evaporation windows. **(d)** Al pads evaporation and lifting off. **(e)** Patterning of $Ge_2Sb_2Te_5$ (GST) sputtering windows on the doped waveguides. **(f)** Sputtering GST layer and its SiO_2 capping, and last lifting off.

Supplementary Figure 2 shows optical microscopic images of our devices in each fabrication step. It includes patterning of SOI waveguides using electron beam lithography (EBL) (JEOL JBX-5500 50kV) and reactive ion etching (RIE) (Oxford Instrument PlasmaPro), and patterning of ion-implantation windows (Supplementary Figure 2a). Next, we performed ion-implantation, photoresist stripping, rapid thermal annealing (RTA) (Jipelec Jetfirst) for dopant activation (Supplementary Figure 2b). The third step of EBL was used for patterning thermal evaporation windows (Supplementary Figure 2c). Al pads were evaporated and lifted off to form Ohmic contact between Al pads and doped silicon (Supplementary Figure 2d). We performed the fourth step of EBL for patterning GST sputtering windows on the doped waveguides (Supplementary Figure 2e). Lastly, GST and its protection layer SiO_2 thin films were sputtered and lifted off (Supplementary Figure 2f).

Supplementary Note 3: Detailed structural parameters of device design

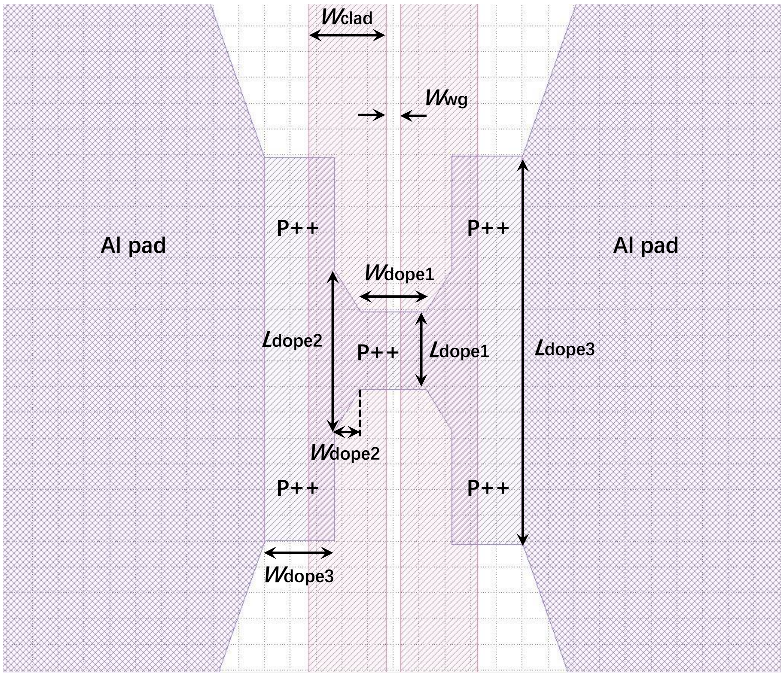

Supplementary Figure 3 GDS design of a SOI waveguide microheater with labeled structural parameters.

GDS design of a SOI waveguide microheater is shown in Supplementary Figure 3 with labelled and defined structural parameters: L_{dope3} = 15 µm, W_{dope3} = 2.7 µm, W_{dope2} = 1 µm, W_{dope1} = 2.5 µm, W_{wg} = 0.55 µm, W_{clad} = 3 µm, L_{dope2} = L_{dope1} + 3.2 µm and L_{dope1} is varied between 1 and 15 µm.

Supplementary Note 4: Setup for electrical programming and *in situ* optical probing

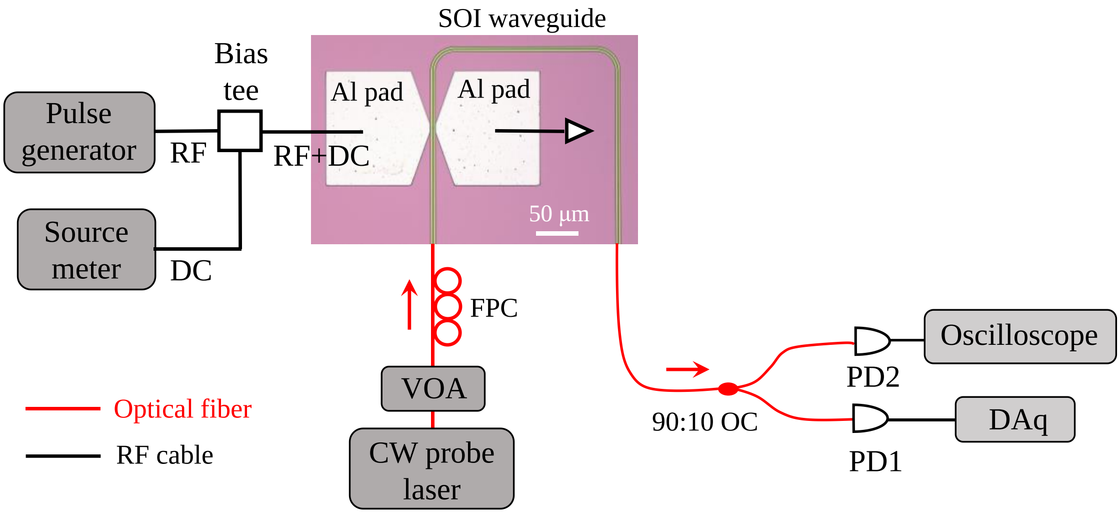

Supplementary Figure 4 Experimental setup for electrical programming and *in situ* optical probing of GST cells. RF, radio frequency. DC, direct current. FPC, fiber polarization controller. CW, continuous wave. OC, optical coupler. PD, photodetector. DAq, data acquisition.

Supplementary Figure 4 shows an experimental setup for investigating temporal response of a GST cell for binary operation, multilevel operation, and scalar multiplication $c = a \times b$. A source meter (Keithley 2614B) was used for current-voltage scanning. Short electrical pulses were generated from an electric pulse generator (Tektronix AFG3102C) for pulse amplitude modulation (PAM) (0−10V) and pulse width modulation (PWM). RF and DC electrical signals were combined by a bias tee (Mini-Circuits ZFBT-4R2GW+) and were sent into devices by contacting a GS probe with two Al pads of a microheater. CW probe light at a wavelength of 1570.4 nm and a power of 3.55 mW (7711A, Keysight Technologies) was sent to a variable optical attenuator (Thorlabs V1550A) for data encoding in demonstrating scalar multiplication operation. Transmission of the fundamental transverse-electric (TE) mode was optimized by a fiber polarization controller (FPC) (Thorlabs, FPC032). Device output was split into two paths for light detection by using a low-speed and low-noise photodetector (Newport 2011-FC) and a high-speed photodetector (Newport 1811-FC), which respectively detect temporal binary/multilevel response and switching dynamics of a GST cell.

Supplementary Note 5: Measured optical loss due to ion implantation

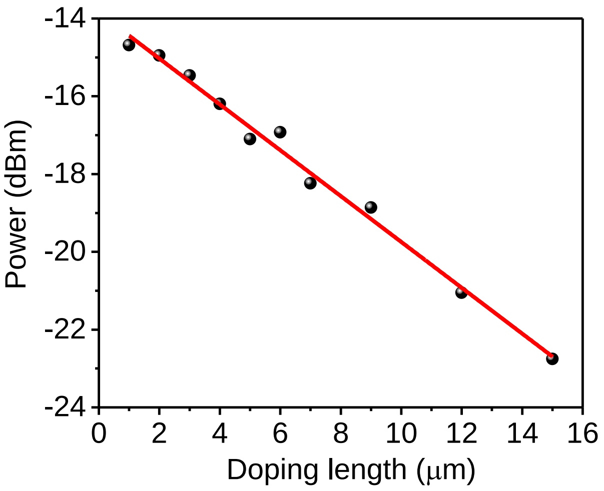

Supplementary Figure 5 Measured output power versus doping length of waveguide microheaters.

For an array of fabricated waveguide devices, we only varied doping length while the rest geometric parameters were designed identically. And we measured output power of these devices. Supplementary Figure 5 shows measured output power versus doping length ranging from 1 to 15 μm. These data were linearly fitted with a fitted loss of 0.59 dB/μm due to free carrier absorption in heavily doped silicon.

Supplementary Note 6: Numerical simulation of electro-thermal profiles

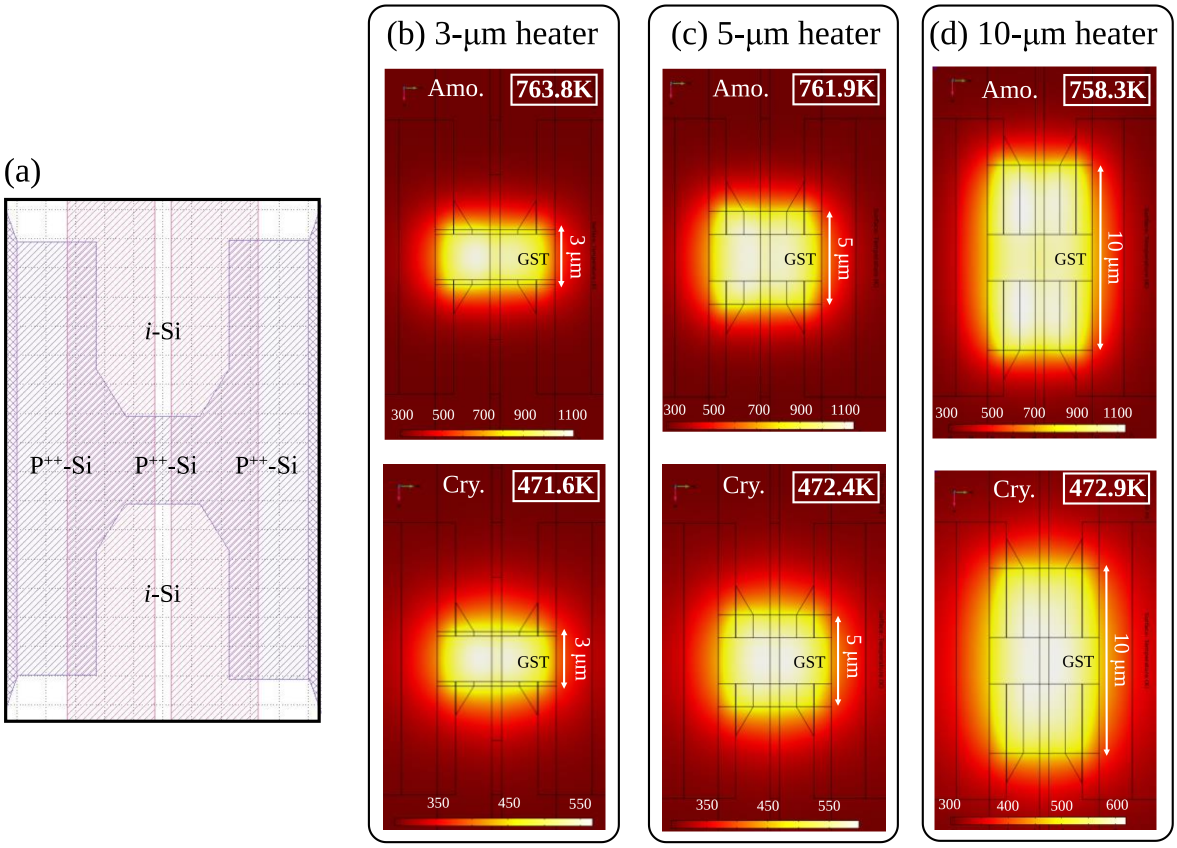

Supplementary Figure 6 Numerical investigation of doping length dependent thermal profiles. (a) Schematic of a SOI waveguide microheater with labeled P^{++} doping region (P^{++}–Si) and undoped intrinsic silicon (*i*–Si). **(b)–(d)** Simulated thermal profiles of microheaters at end of amorphization (pulse width = 50 ns and pulse amplitude = 7 V) and crystallization electrical pulses (pulse width = 200 ns and pulse amplitude = 3 V) with doping lengths of 3 μm (b), 5 μm (c), 10 μm (d). Length of a GST patch along the waveguide is fixed at 2.5 μm. The lowest temperature in each doped waveguide rib is shown in the top right rectangular box. Amo.: amorphization and Cry.: crystallization.

We developed a 3D finite element (FE) model with coupled electro-thermal interactions in our devices using COMSOL Multiphysics. Schematic of the model is consistent with structure of the actual device. Supplementary Table 1 lists the material properties used in our simulations. Thermal boundary resistance was applied to all the internal boundaries. Electrically insulating boundary conditions were applied on all external boundaries except those across the electrodes, where a current flow was applied. In the simulation, we sent 50-ns 7V amorphization pulse and 200-ns 3V crystallization pulse. At the end of pulse heating, thermal profiles of devices were recorded and shown in Supplementary Figure 6. The lowest temperature in each doped waveguide rib is also labeled. Heating area expands with increasing doping length along the waveguide, which can be used for switching a larger area of GST. Especially, glass transition temperature and melting temperature of the GST are $T_x = 433$ K and $T_m = 817$ K, respectively.[1]

The maximal temperatures of GST cells in Supplementary Figure 6b–6d all surpass T_m (surpass T_x and below T_m) to achieve amorphization (recrystallization).

Supplementary Table 1 Material properties.

Material	Heat capacity (J/(kg·K))	Thermal conductivity (W/(m·K))	Electric conductivity (S/m)	Density (kg/m^3)
Al	900	238	3.77×10^7	2700
cGST [1]	212	$0.19(\frac{T}{300})^{2.27}$	[2]	6300
P^{++} Silicon [3]	720	149	[4-8]	2330
SiO_2 [1]	709	1.38	10^{-14}	2203

Note: Material properties of Al was taken from the default database of COMSOL Multiphysics while other materials' properties were from literature as denoted.

Supplementary Note 7: Repetitive switching of our GST cells

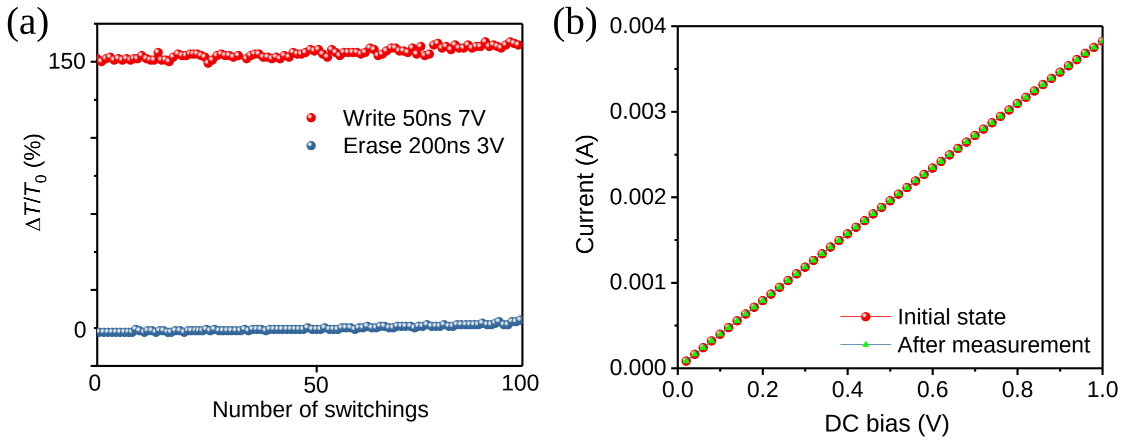

Supplementary Figure 7 Repetitive switching of a GST cell based on the SOI waveguide microheater. (a) Repetitive optical switching between amorphous and fully crystalline states over 100 cycles. **(b)** Repetitive IV scanning.

Supplementary Figure 7a shows operation of our device with repetitive binary electrical switching over 100 cycles with a large switching contrast of 150%. Write and Erase states of the GST cell were programmed by a single 50-ns and 7V electrical pulse and a single 200-ns and 3V electrical pulse, respectively. Before and after repetitive switching, we performed IV scan and found there is no variation in the total resistance of our microheater, which guarantees a robust operation for pulse heating.

Please note that there are drifts in transmission as shown in Supplementary Figure 7a. Drift in transmission during the cycling test was observed in GST-cladded waveguide microheaters based on different designs, e.g., the P-type doped[9] and PIN diode microheaters.[10] Mechanisms of drift were attributed to material degradation and thermal reflowing of the GST thin film after multiple switching cycles.[10]

To bypass the drift issue, recalibration is used to maintain the programming consistency and normal operation of our devices. Recalibration was done by calculating $[(T-T_{base})/(T_{max}-T_{base})]$, where T_{max} and T_{base} (T_0) were updated in our real-time measurement during PCM switching.

For a more general solution to avoid phase segregation, a phase-change heterostructure (PCH) could possibly be applied for photonic computing. Ding *et al.* reported alternately grown thin films with 5 nm Sb_2Te_3 and 3 nm $TiTe_2$.[11] The crystalline $TiTe_2$ nanolayers remained robust while the Sb_2Te_3 nanolayers were switched between the crystalline and amorphous phase. The strong confinement effects of $TiTe_2$ walls suppressed resistance drift and phase separation tendency in electronic devices, which resulted in a much higher programming consistency and a much improved cycling endurance. Recently, it has predicted a sizable change in optical properties upon phase transition in PCH by ab initio simulations.[12] Hence, PCH could be a promising candidate to replace GST to alleviate the transmission drift of optical devices. In addition, a monatomic PCM (pure antimony) has also been reported for photonic programming.[13] The lifetime of amorphous antimony is improved by scaling down the film thickness to 5 nm and below. Future work utilizing new PCMs for photonic computing is thus anticipated.

Supplementary Note 8: Performance comparison of non-resonant straight waveguide memory cells

Supplementary Table 2 Performance comparison of the non-volatile electrically reprogrammable non-resonant straight waveguide memory cells based on GST.

Microheater devices	Crystallization		Amorphization		Modulation depth (dB)	Number of levels	Refs
	Energy (nJ)	Energy consumption/ modulation depth (nJ/dB)	Energy (nJ)	Energy consumption/ modulation depth (nJ/dB)			
P^{++} doped Si heater	9	1.7	10	1.8	5.44	5	9
PIN diode Si coupler heater	6830	683	380	38	10	2	10
PIN diode Si heater	715	406.3	13	7.4	1.76	2	14
Graphene heater	860.7	286.9	5.55	1.8	3	7	15
Plasmonic nanogap heater	4	8.9	0.65	1.4	0.45	6	16
P^{++} doped Si heater	6.88	1.7	8.84	2.1	4.13	18	This work

Supplementary Table 2 compares performances of our experimentally demonstrated GST cells with the previously reported non-resonant straight waveguide GST cells, and shows that our GST cells exhibit the highest encoding levels reported so far and the lowest energy consumption per unit modulation depth in the crystallization process. We also note that storage multilevel may be enhanced by incorporating a GST cell into a resonator,[17] however, it has a reduced compute density due to resonance-induced narrower bandwidth for WDM-based parallel photonic computing.

Supplementary Note 9: Calibration on normalized transmission of a VOA and a GST cell

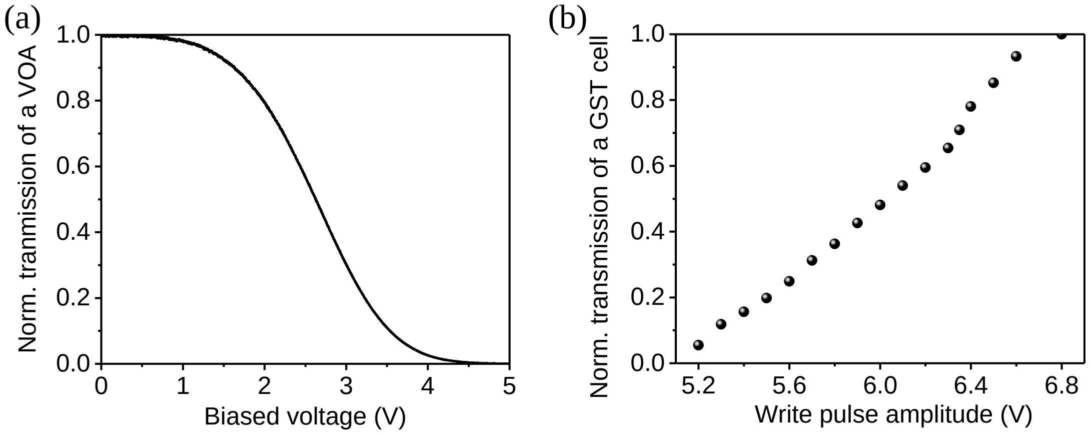

Supplementary Figure 8 Normalized transmission of a VOA and a GST cell. (a) Calibrated and normalized input power (P_{in}/P_{max}) of a VOA versus bias voltage. **(b)** Recoded transmittance $(T-T_{base})/(T_{max}-T_{base})$ of a GST cell versus WRITE pulse amplitude with a fixed pulse width of 50 ns.

To calculate the exact result of $x \times w$ (x and $w \in [0, 1]$), input power of a VOA (P_{in}/P_{max}) versus bias voltage (0–5 V) was calibrated and rescaled to [0, 1] to map the multiplicand x, and transmission of a GST cell $[(T-T_{base})/(T_{max}-T_{base})]$ versus Write pulse amplitude (5.2–6.8 V) was recorded and rescaled to [0, 1] to map the multiplier w as shown in Supplementary Figure 8, where P_{max} is the maximal input power encoded by a VOA, T_{base} and T_{max} are respectively the minimal and maximal transmittances of a GST cell programmed electrically.

Supplementary Note 10: Offset correction: post subtraction vs balanced photodetection

Subtraction should be performed due to the non-zero transmission at the lowest level. Taking a simplest case of scalar multiplication for example, x is encoded in the input transmission by mapping x to $\frac{P_{in}}{P_{max}} \in [0,1]$; w is encoded in the transmission of the PCM cell by mapping w to $\frac{T-T_{base}}{T_{max}-T_{base}}$. Therefore, $x \times w$ can be expressed as: $(P_{in} \cdot T - P_{in} \cdot T_{base})/(P_{max} \cdot T_{max} - P_{max} \cdot T_{base})$, in which absolute transmission through the device including $P_{in} \cdot T$, $P_{in} \cdot T_{base}$, $P_{max} \cdot T_{max}$, $P_{max} \cdot T_{base}$ were recorded. The two terms related to P_{max} only need to be measured once since P_{max} is fixed. The other two terms related to P_{in} should be measured for every digital input value. The subtraction operation of $(P_{in} \cdot T - P_{in} \cdot T_{base})$ was post-processed on a digital computer to correct offset.

Alternatively, as a more efficient method, one can implement this offset correction by using the balanced photodetection method to reduce the load of post processing. Specifically, input

light with a power of $2 \cdot P_{in}$ can be first equally split using a 3-dB power splitter, and then are sent to cells on two different waveguides with transmittance of T and T_{base}, respectively. Last, two outputs are detected simultaneously by a balanced photodetector to generate a photocurrent $(i_+ - i_-)$, where $i_+ = R \cdot (P_{in} \cdot T)$, $i_- = R \cdot (P_{in} \cdot T_{base})$, and R is the responsivity of a photodiode.

<u>Signal processing time:</u>

Compared with the balanced photodetection as shown in Supplementary Figure 9a, the approach using one photodiode in Supplementary Figure 9b doubles signal processing time for each offset correction operation (estimated as: 18.87+250+8×(133.21+68.77) = 1.88 ns) in the current amplification, analog-to-digital conversion, and 8-bit digital data storage in a memory unit. It takes an additional 20 ns for performing a subtraction operation in a floating point unit (FPU).[18]

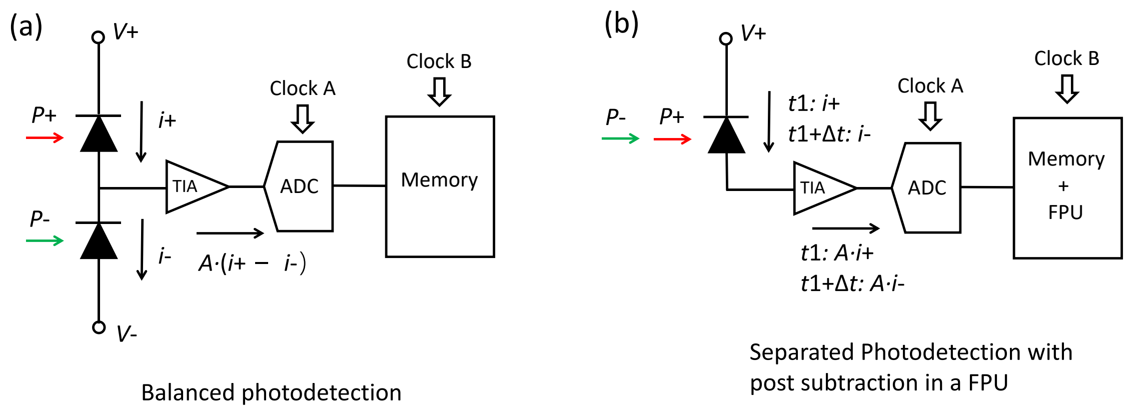

Supplementary Figure 9 Photodetection and signal processing. (a) Schematic of the balanced photodetection. **(b)** Schematic of separated photodetection with a post subtraction in a floating point unit (FPU). PD: photodiode, TIA: transimpedance amplifier, ADC: analog-to-digital converter, FPU: floating point unit.

<u>Energy consumption:</u>

Energy consumption can be expressed as $E = (V_+ \cdot I_+ + |V_-| \cdot I_-) / B_{data}$ for a balanced PD and $E = (V_+ \cdot I_+ + V_+ \cdot I_-) / B_{data}$ for one PD with two separated detection. For these two scenarios, energy consumptions are the same if $V_+ = - V_-$, where $V_\pm$ is the bias voltage, $I_\pm$ is the photocurrent, and B_{data} is the data rate.[19,20]

Besides, compared with the balanced photodetection, the approach using one photodiode requires more energy in powering of a transimpedance amplifier (TIA), an analog-to-digital converter (ADC), a memory unit, and a FPU. Additional energy consumption is estimated as (0.65+22+0.041+10.25) = 32.94 pJ for each offset correction operation. Some more details can be found in Supplementary Table 3.

Supplementary Table 3 Estimated energy consumption and operating time of components in the signal processing circuits.

Components	energy consumption	Operating time (Clock frequency/sampling rate)	Reference
TIA	0.65 pJ/bit	18.87 ps (53 Gbit/s)	21
8-bit ADC	88(mW)/(4 Gsamples/s) = 22 pJ	250 ps (4 Gsamples/s)	22
Memory unit	Write: 3700 aJ<br>Read: 1500 aJ<br>$8 \times (3700+1500) \times 10^{-6}$ = 0.041 (pJ) (8-bit digital data)	Write: 133.21 ps<br>Read: 68.77 ps<br>8×(133.21+68.77)=1615.84 ps (8-bit digital data)	23
FPU	10.25 pJ/operation	5×4 = 20 ns (delivering each result after 4 clock cycles with a clock period of 5 ns)	18

Supplementary Note 11: Nearly identical switching contrast probed for WDM light

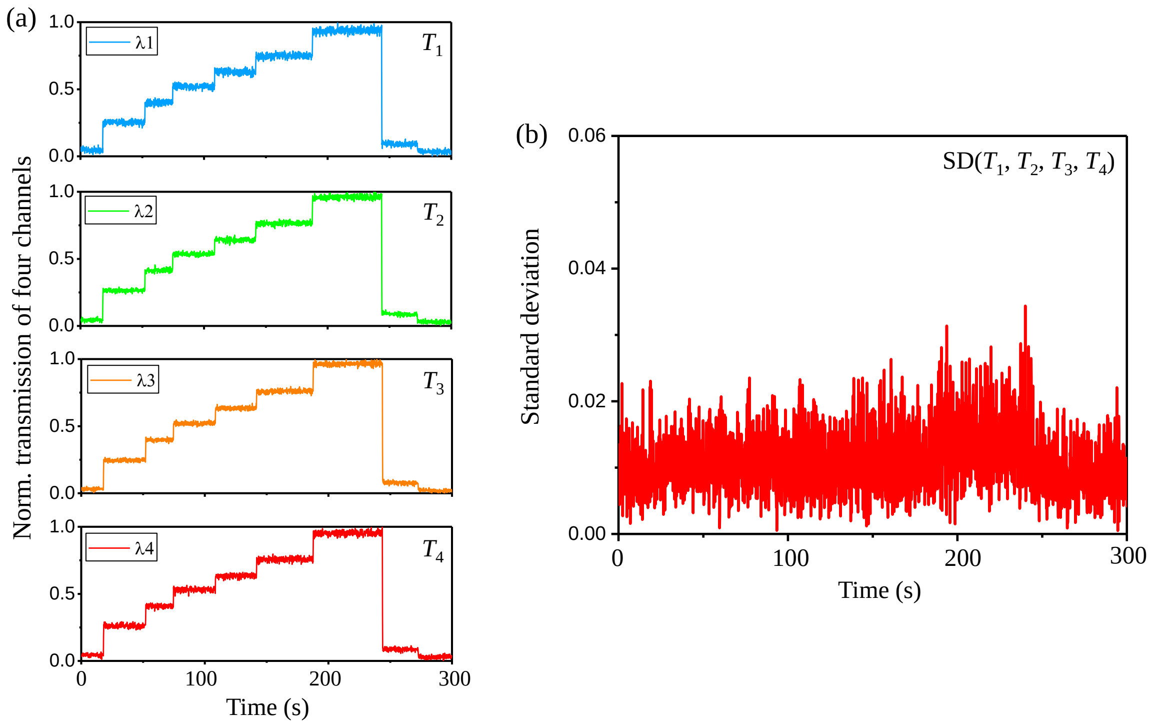

Supplementary Figure 10 Nearly identical switching contrast probed for wavelength multiplexed light passing through a GST cell. (a) Normalized transmission of four channels versus eight switching events. **(b)** Standard deviation of the normalized transmission of four channels.

In principle, light multiplexed in one waveguide should be probed with the same switching contrast $\Delta T/T_{base}$ upon electrical switching of a GST cell. To validate, we recorded and normalized transmission of four parallel channels versus eight switching events with random increments in transmission levels as shown in Supplementary Figure 10a, and calculated standard deviation $SD(T_1, T_2, T_3, T_4)$ of four channels versus time as shown in Supplementary Figure 10b. Averaged standard deviation was calculated as 1.10%, which may be due to photodetection noise.

Supplementary Note 12: Photodetection noise, bandwidth and light source power drift

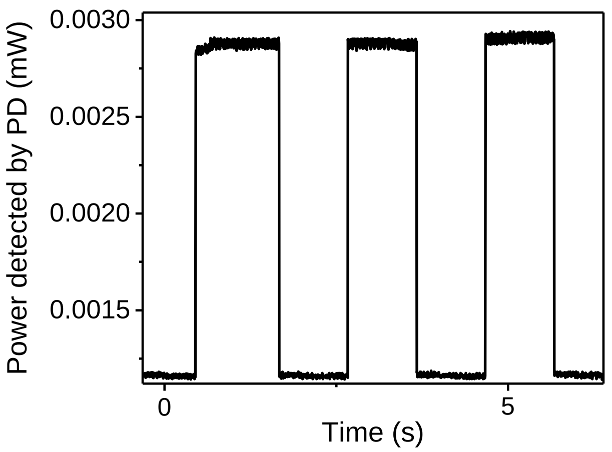

Supplementary Figure 11 Photodetection noise.

As shown in Supplementary Figure 11, standard deviation of our measured power fluctuation (noise) in the detected probe light is 10.2 (16.8) nW at a detected power of 1.19 (2.91) μW. Thus, the noise is contributed by the shot noise and thermal noise ($i^2_{\mathrm{shot}} + i^2_{\mathrm{thermal}}$) for our photodetector. We also note that $i^2_{\mathrm{noise}} = i^2_{\mathrm{shot}} + i^2_{\mathrm{thermal}} = 2qI_{\mathrm{ph}}B + 4kTB/R$, which is linearly proportional to the I_{ph} if the temperature T is fixed at the room temperature. Thus, detected power contributed by the thermal noise is fitted as 5.63 nW.

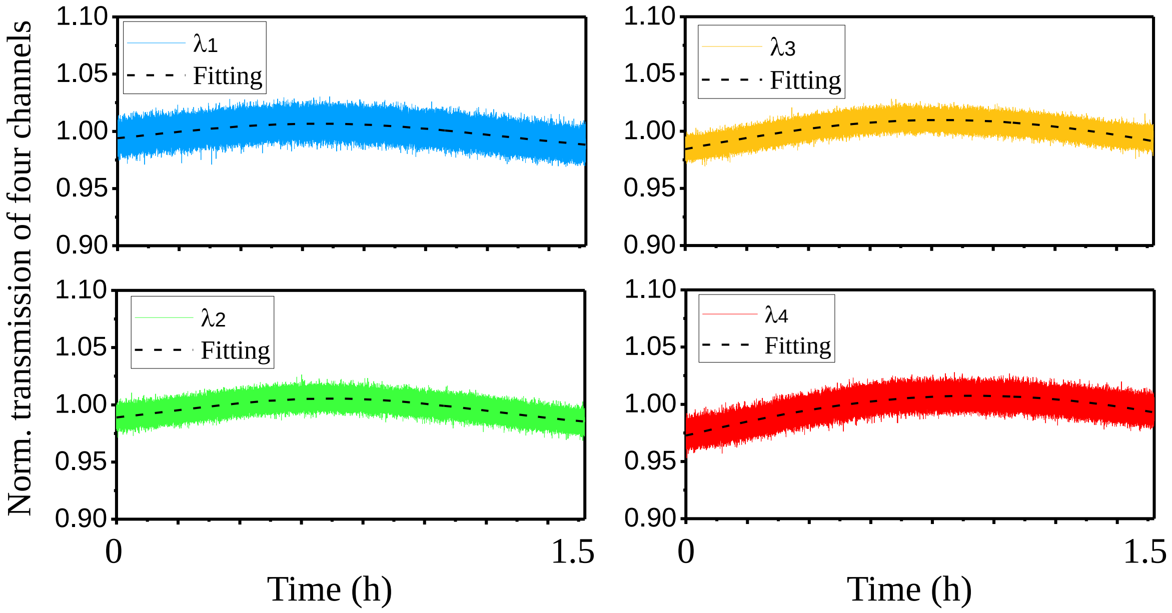

Supplementary Figure 12 Monitored normalized transmission of four channels within 1.5 hours for calculating photodetection noise in transmission.

Output light in the four wavelength channels of the photonic chip were recorded for 1.5 hours. Noise from a photodetector is defined as: SD(T) (unit:%), where T is the detected and normalized transmission of each channel as shown in Supplementary Figure 12. Here, normalization was performed with respect to the average transmission. And noise of photodetectors from channel 1 to 4 were calculated to be 0.79%, 0.74%, 0.81%, and 1.07%, respectively, which are below or around 1%.

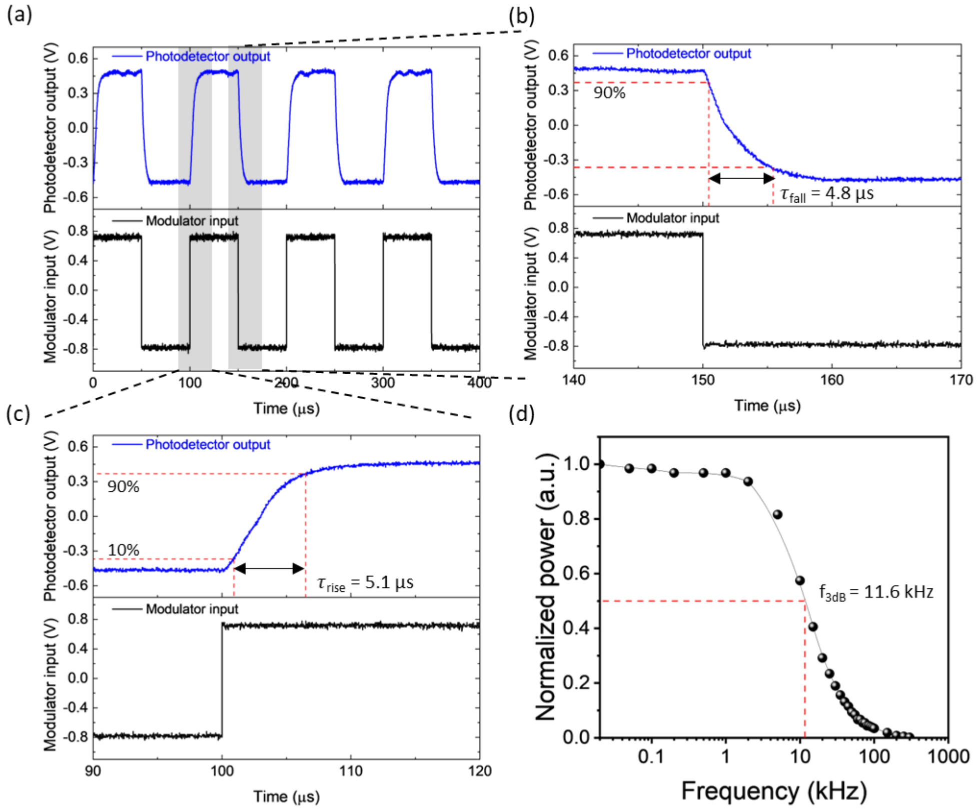

Supplementary Figure 13 Investigation on the photodetector bandwidth. The low-pass filter of a photodetector (Newport 2011-FC) is set to 10 kHz. **(a)** Photodetector output of square-wave modulated light. **(b)** Zoom-in of (a) showing 4.8-μs fall time. **(c)** Zoom-in of (a) showing 5.1-μs rise time. **(d)** 11.6-kHz 3-dB bandwidth of the photodetector.

Since the speed of a VOA is 1 kHz, we set the low-pass filter of a photodetector at 10 kHz. The output waveform (converted to voltage by transimpedance amplifier) is presented in Supplementary Figure 13. The fall time and rise time of the photodetector are 4.8 μs and 5.1 μs, respectively. The 3 dB-bandwidth of the photodetector is measured as 11.6 kHz as shown in Supplementary Figure 13d, which is close to the set value of 10 kHz.

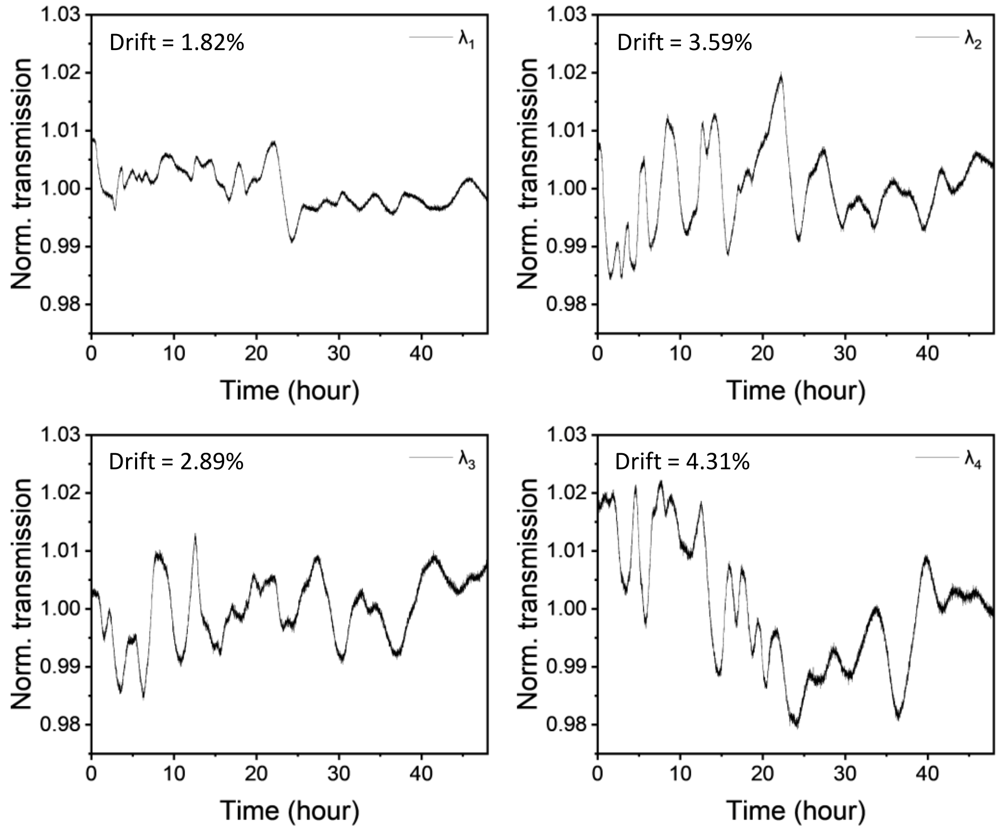

Supplementary Figure 14 Monitoring power drift of the four wavelength channels of our broadband light source for two days. The drifts are respectively 1.82%, 3.59%, 2.89%, and 4.31% from the four channels.

In order to investigate power drift of the light source, we monitored the transmission of four wavelength channels for 2 days. A wavelength demultiplexer was used to separate the four wavelength channels from a broadband light source, and power of each channel was recorded by a photodetector. Down sampling was performed through recording transmission by every 20 seconds. The results are shown in Supplementary Figure 14. Without light coupling into and out of a photonic chip (coupling loss ~ 20 dB), the signal-to-noise ratio is much higher than those as shown in Supplementary Figure 12. Drift of each channel can be defined as: $2\cdot[\mathrm{Max}(T)-\mathrm{Min}(T)]/[\mathrm{Max}(T)+\mathrm{Min}(T)]$ (unit:%), where Max(T) and Min(T) are the maximal and minimal values of the normalized transmission (black dashed curves). Here, normalization was performed with respect to the average transmission. And drift of channel 1 to 4 were calculated to be 1.82%, 3.59%, 2.89%, and 4.31%, respectively. It may impose large computation errors when photodetection noise and drift are comparable with the switching contrasts. However, computation error can be largely suppressed with an enhanced contrast-to-noise ratio (CNR). We also note that measured drift of our used broadband light source (1.82%–4.31%) is higher than our used CW probe laser (0.7%) for performing scalar multiplication as shown in Figure 2d.[24]

Supplementary Note 13: Boxcar averaging and error distributions of the three color tones

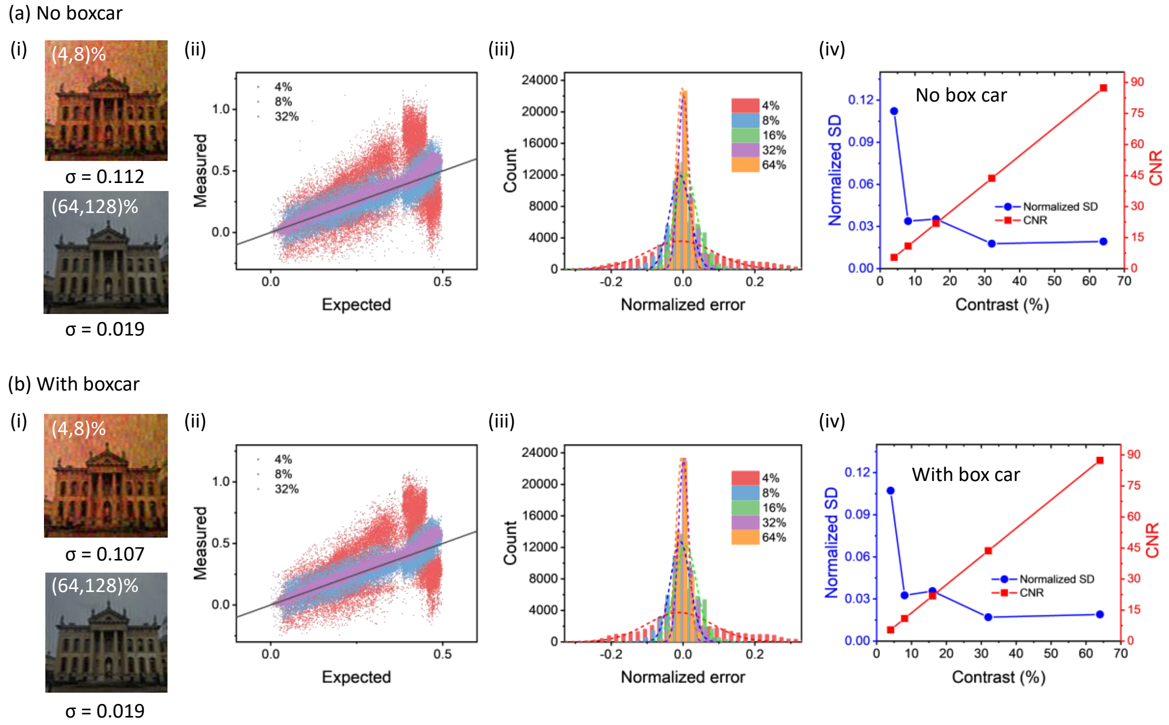

Supplementary Figure 15 Image brightness scaling results (scaling factor = 0.5) using different switching contrasts ($\Delta T_1/T_{base}$) without and with boxcar averaging. (a) Without boxcar averaging. **(b)** With boxcar averaging. The processed images with labelled SDs using the lowest and highest switching contrasts as shown in (i) with statistical analysis on the accuracy plots (ii), error distributions (iii), normalized SD and CNR (iv) versus the contrast ($\Delta T_1/T_{base}$).

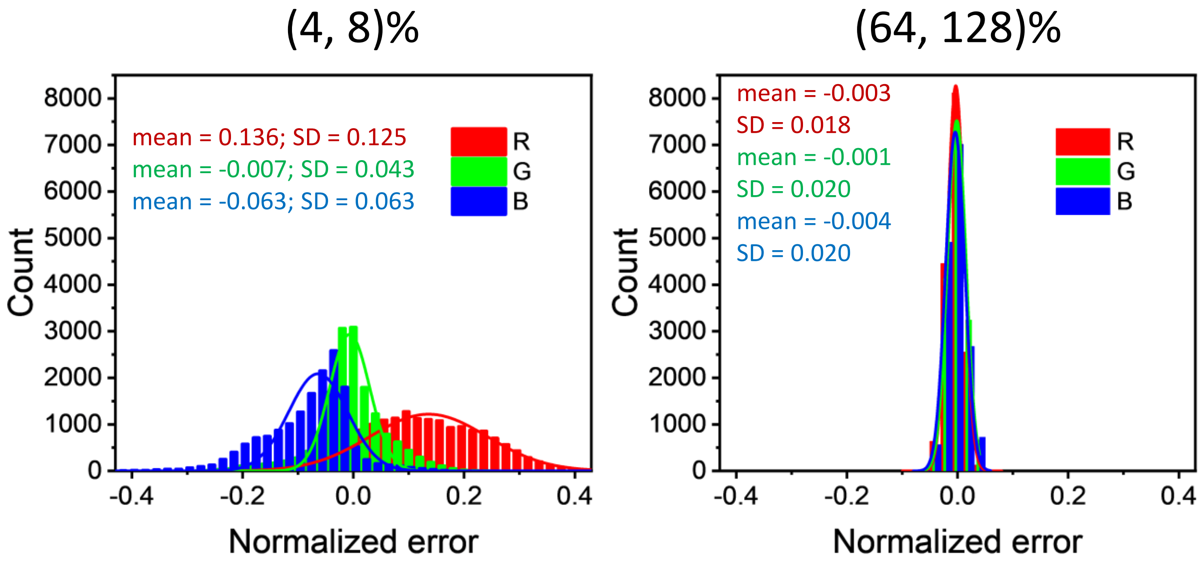

Supplementary Figure 16 Investigation on the error distributions of the three color tones (RGB). At the low switching contrasts (4, 8)%, change of hue can be observed with higher

noise. At the high switching contrasts (64, 128)%, change of hue is not observed and lower noise is achieved.

We performed boxcar averaging (averaging five sample points) to confirm the change of hue is not caused by photodetection noise. Instead, the change of hue is due to system error. Supplementary Figure 15 shows the brightness scaling results (scaling factor = 0.5) without and with boxcar averaging in Supplementary Figure 15a and Supplementary Figure 15b, respectively. The two results are almost identical as revealed by the labelled SDs, accuracy plots in (ii), error distributions in (iii), and normalized SD and CNR in (iv) versus the contrast ($\Delta T_1/T_{\text{base}}$). These results show that photodetection noise is not causing change of hue, because otherwise the boxcar averaging will reduce it. We also plotted the error distributions of three color tones (RGB). Their error distributions are shown in Supplementary Figure 16. When the switching contrasts are low at (4, 8)%, the low computing accuracy leads to shifted normal distributions. Their centers are shifted to 0.136, −0.007, and −0.063 for the RGB color tones, respectively. This explains why the image looks red. However, the change of hue is not observed when the switching contrasts are large at (64, 128)% as shown in Supplementary Figure 15i and Supplementary Figure 16. The improvement is attributed to the electrical switching using microheaters that provide higher switching contrasts to minimize the impact of laser power drift.

Supplementary Note 14: WDM enabled parallel multiplication for image processing

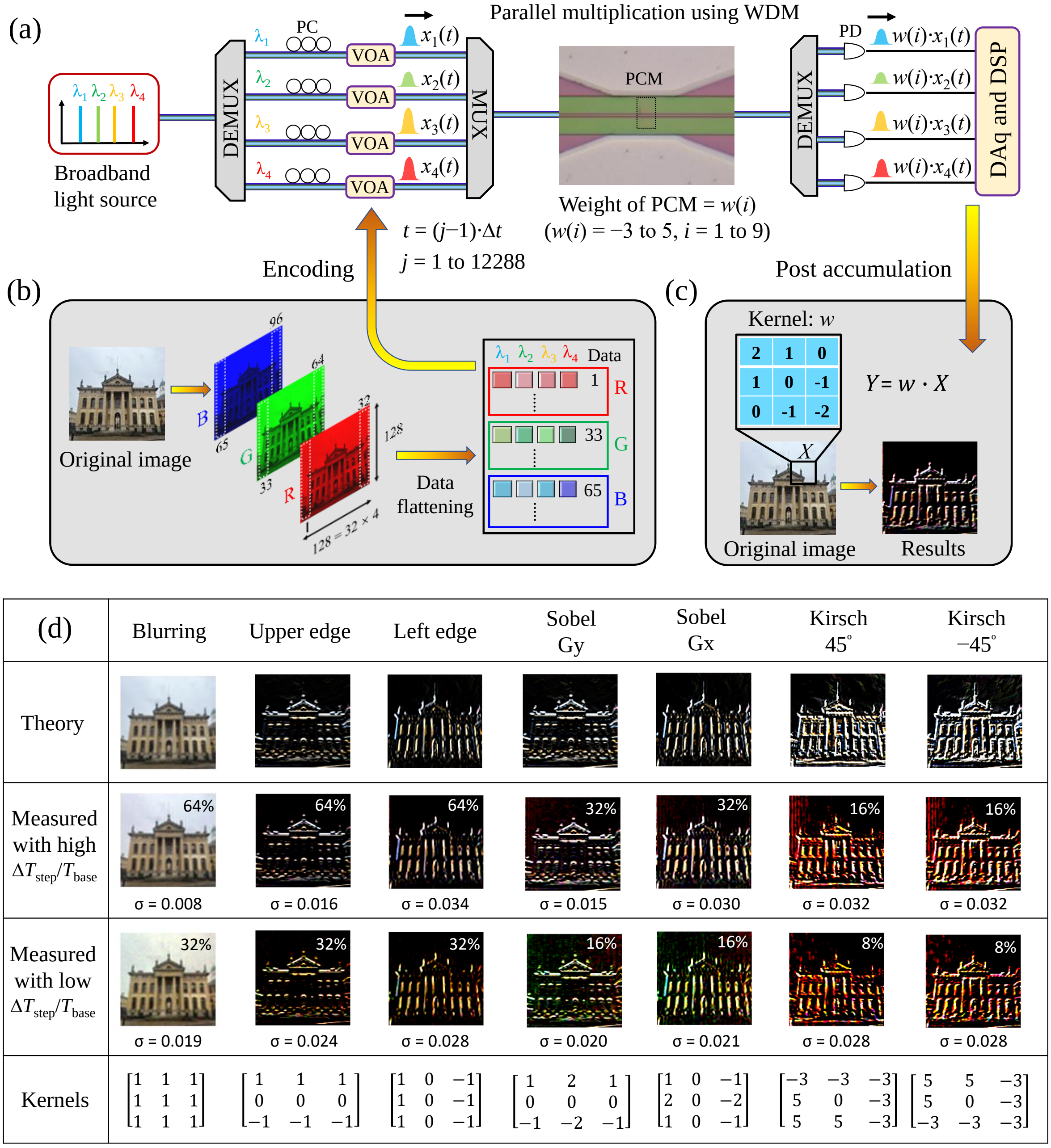

Supplementary Figure 17 WDM enabled parallel multiplication for image processing. (a) An experimental setup for parallel multiplication. **(b)** Flattened pixel data of an RGB image for data encoding. **(c)** Post accumulation of stored multiplication results for convolutional operation with an example showing upper and left edges detection by a 3×3 kernel matrix. (The original image showing the Clarendon Building in the University of Oxford was photographed by authors). **(d)** Processed images by applying seven different image kernels with high and low $\Delta T_{step}/T_{base}$.

We exploited parallel multiplication for realizing advanced image filtering. Please note that we performed in-memory scalar multiplication using a GST cell and software post accumulation to complete convolutional operation. As shown in Supplementary Figure 17a and 17b, we used the same experimental setup in Figure 3a and data flattening process. However, parallel scalar multiplication process of an entire RGB image was repeated for all kernel elements with multiplication results stored for software post accumulation to complete convolutional operation. Except kernels with identical elements (e.g., the blurring kernel), transmission of a GST cell was programmed as $T(w(i)) = T_{\text{base}} + [w(i) - \min(w(i))] \cdot (T_{max} - T_{\text{base}}) / [\max(w(i)) - \min(w(i))]$, where $\min(w(i))$ and $\max(w(i))$ are the minimum and maximum values of kernel elements, respectively. And we denote $\Delta T_{\text{step}} = (T_{\max} - T_{\text{base}}) / [\max(w(i)) - \min(w(i))]$, and $\Delta T_{\text{step}}/T_{\text{base}}$ is an incremental step of switching contrast in partial amorphization of a GST cell from its baseline. As an example, it has 5 different elements ($w(i) = \pm2, \pm1$, and 0) in a kernel w for extracting the upper and left edges as shown in Supplementary Figure 17c. Transmission of a GST cell was programmed as $T(w(i)) = T_{\text{base}} + (w(i) + 2) \cdot (T_{\max} - T_{\text{base}})/4$, where $\min(w(i)) = -2$ and $\max(w(i)) = 2$, and $\Delta T_{\text{step}}/T_{\text{base}} = 32\%$. Thus, $T(w(i))$ of a GST cell was programmed as $T_{\text{base}} + 0.32 \cdot (w(i) + 2) \cdot T_{\text{base}}$ in five switching events. After each switching event, we performed scalar multiplication of an entire RGB image with the GST cell with a transmission of $T(w(i))$. After software accumulation, the upper and left edge features are clearly visible, which validates the effectiveness of our convolution. In a next step, a blurring kernel with six identical elements ($w(i) = 1$, $i = 1$ to 9) was implemented at two different transmission levels of a GST cell ($T = T_{\text{base}} + \Delta T_{\text{step}}$ and $\Delta T_{\text{step}}/T_{\text{base}} = 64\%$ and 32%). Measured result with a higher $\Delta T_{\text{step}}/T_{\text{base}}$ (64%) matches well with the theoretical result due to a better CNR and a relative smaller computation error (SD = 0.008), however, the measured result shows change of hue in the sky background with $\Delta T_{\text{step}}/T_{\text{base}} = 32\%$. Computing error is mainly from photodetection noise and power drift due to instability of our broadband light source (Supplementary Figure 12 and Supplementary Figure 14). Similarly, it exhibits good matching between the measured results and theoretical computation for applying kernels of upper/left and Sobel upper/left edges detection with $\Delta T_{\text{step}}/T_{\text{base}} = 64\%$ and 32%. Based on the Gaussian fitting of computing errors, their SDs are between 0.015 and 0.034. Although edge features of Sobel and Kirsch $\pm45^{\circ}$ kernels are clearly extracted with $\Delta T_{\text{step}}/T_{\text{base}} = 16\%$, snow noise and stripe noise imposed on the background are visible. Obviously, large switch contrasts ($\Delta T_{\text{step}}/T_{\text{base}}$) achieved by electrical programming of GST cells using microheaters are crucial for advanced image processing and feature extraction for CNNs visualized from the above comparison as shown in Supplementary Figure 17d. And $\Delta T_{\text{step}}/T_{\text{base}}$ higher than 32% maybe required to obtain good quality of a processed image based on our experimental setup. Thus, the full switching contrast larger than 256% (8·32%) may be required for applying the Kirsch filter. It is possible to further improve the switching contrast by optimizing the microheater design. In one study, the full switching contrast was reported as 400% using a doped waveguide crossing structure.[9] In contrast, previously demonstrated SOI waveguide memory cells using the all-optical programming suffer from low contrasts (with a full contrast of merely 15%).[25] It would be difficult for them to obtain good image processing results due to limited $\Delta T_{\text{step}}/T_{\text{base}}$ less than 16%.

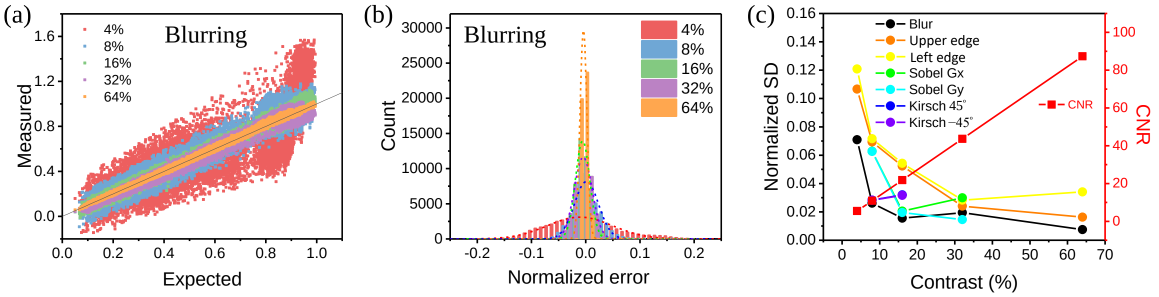

Supplementary Figure 18 Computational precisions for image processing. (a) and **(b)** An example of applying blurring kernel with measured scalar multiplication results versus the exact results (a), histograms of normalized error fitted with Gaussian distributions (b). **(c)** Normalized SD and CNR versus switching contrasts of a GST cell for applying blurring, upper edge, left edge, Sobel Gx/Gy, and Kirsch ±45° kernels.

Supplementary Figure 18a shows one example of measured results versus the exact results at switching contrasts $\Delta T_{\text{step}}/T_{\text{base}}$ of 4%, 8%, 16%, 32%, and 64% after applying image blurring. Supplementary Figure 18b shows histograms of computational error calculated by subtracting the measured results from the exact results. Histograms were further fitted by Gaussian distributions to extract SDs. Based on the same process, we calculated SD and CNR of applying various image kernels, and plotted results in Supplementary Figure 18c. We observed the same trend, i. e., SD decreases (CNR increases) with increase of the switching contrast. For examples, SDs are suppressed from (0.071, 0.107, 0.121, 0.063, 0.063) to (0.008, 0.016, 0.028, 0.021, 0.015) for applying (blurring, upper edge, left edge, Sobel Gx, Sobel Gy) kernels by increasing $\Delta T_{\text{step}}/T_{\text{base}}$ from 4% to 64%. SDs were extracted to be 0.028 and 0.028 for applying the Kirsch 45° and −45° kernels, respectively. CNR was improved from 5.46 to 87.36 by increasing switching contrasts $\Delta T_{\text{step}}/T_{\text{base}}$ from 4% to 64%. In addition, the detailed computing accuracies and error distributions generated from applying upper edge, left edge, Sobel Gx, Sobel Gy, Kirsch 45° and Kirsch −45° image kernels are presented in Supplementary Figure 19.

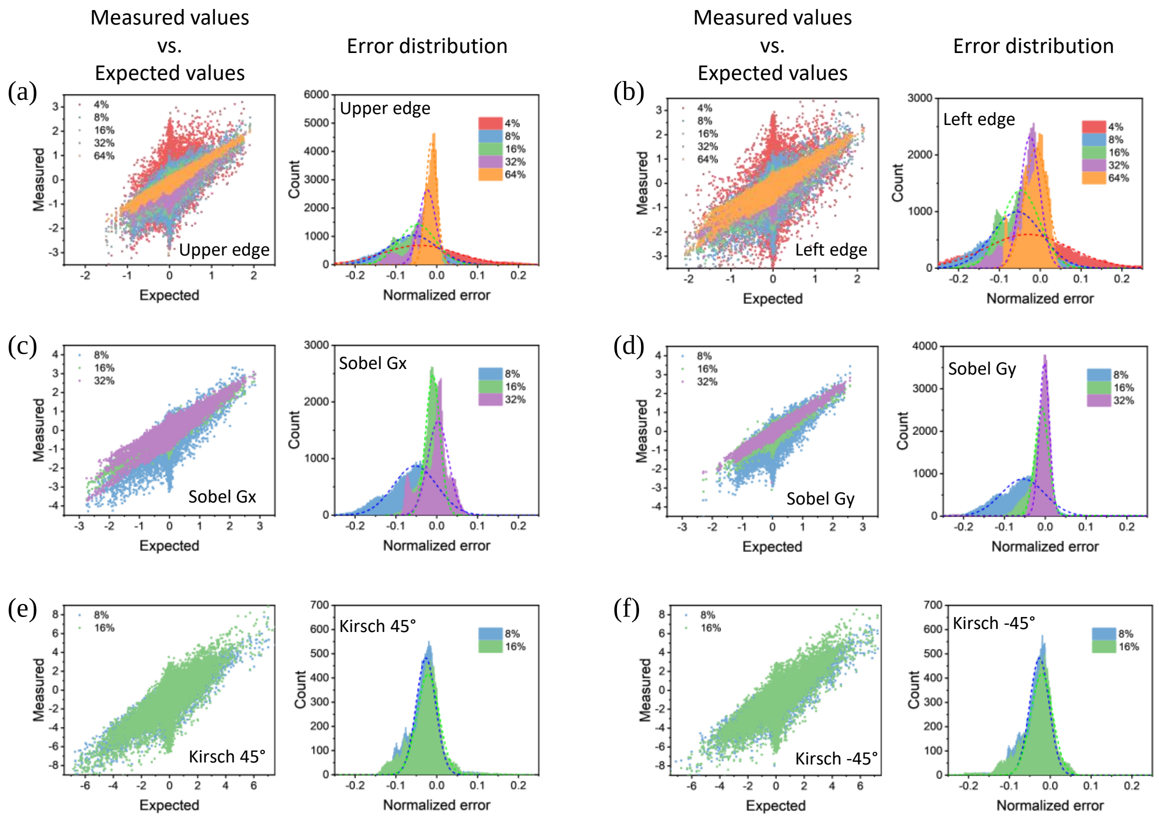

Supplementary Figure 19 Computing accuracies and error distributions versus switching contrasts for (a) upper edge, **(b)** left edge, **(c)** Sobel Gx, **(d)** Sobel Gy, **(e)** Kirsch 45° and **(f)** Kirsch −45° image kernels.

Supplementary Note 15: Computing accuracy of the convolutional layer, training of convolutional neural networks (CNNs) and comparison on prediction accuracies, estimation on computational load

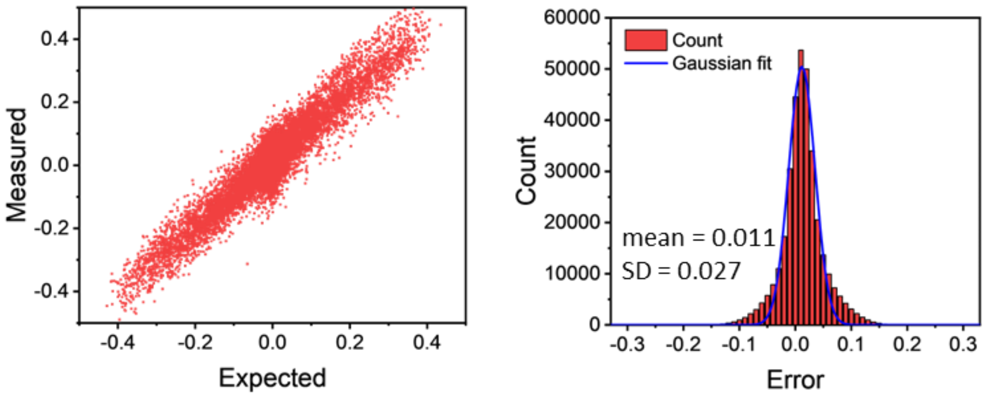

Supplementary Figure 20 Computing accuracy and error distribution of using the photonic dot-product engine for convolutional processing of MNIST fashion products and hand-written digits database.

The convolutional layer is implemented using our photonic processor to generate measured results and using a computer to generate the expected results as to study the accuracy of convolutional processing for MNIST fashion products and hand-written digits database and related error distribution. The results are shown in Supplementary Figure 20. The error distribution is fitted by a Gaussian distribution with the mean error of 0.011 and SD of 0.027.

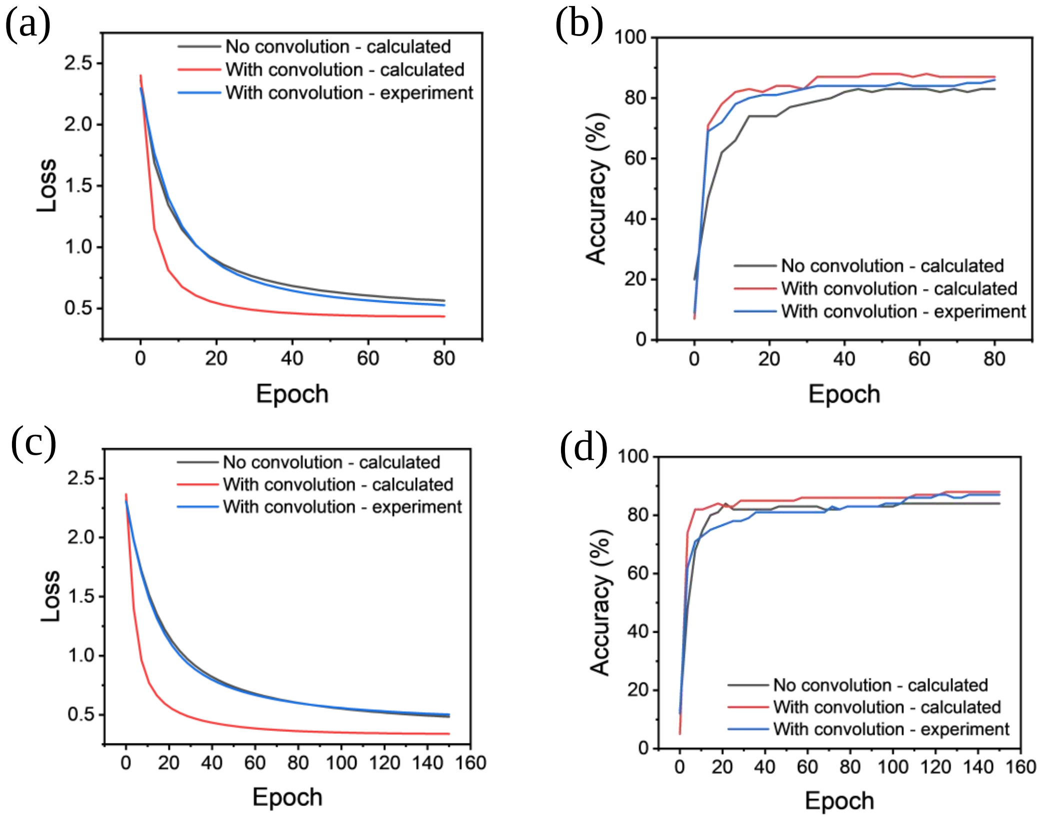

Supplementary Figure 21 Testing loss and accuracy of CNNs versus epoch. (a) and **(b)** Evolution of loss and accuracy for MNIST fashion product recognition. **(c)** and **(d)** Evolution of loss and accuracy for MNIST hand-written digit recognition.

Training of CNN was performed using a digital computer due to lack of on-chip scalable neurons.[26-29] The CNN simulation models were built in MATLAB using the Adam optimizer with default settings except for the number of epochs. Training epochs of 80 and 150 were defined for MNIST fashion product and handwritten digit, respectively, due to saturated loss with increased epochs according to Supplementary Figure 21a and 21c. As shown in Supplementary Figure 21b and 21d, with the help of convolutional layers, prediction accuracies were improved compared with those of the fully connected neural networks without convolutional layers. Based on the experimentally measured data, classification accuracies were tested as 86% and 87% for fashion product and digit, respectively, which agree well with the theoretical calculation results.

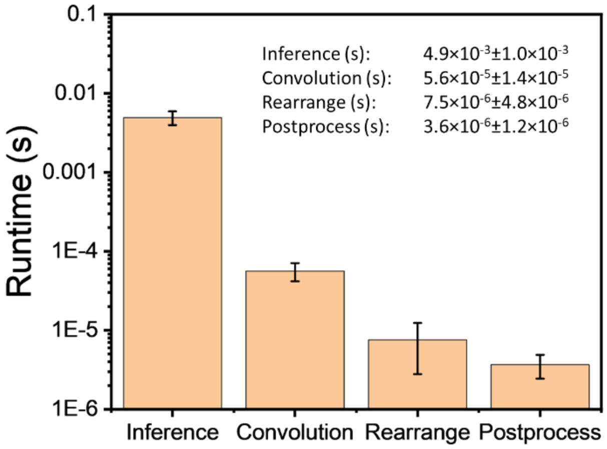

Supplementary Figure 22 Estimation on computational load.

We run inference using the trained neural network (architecture shown in Figure 4b) to estimate the computational load related to optical processing. The results are shown in Supplementary Figure 22. The whole inference step takes 4.9×10^{-3} s. The convolutional processing step takes 5.6×10^{-5} s. Thus, the optical processing contributes 1.1% to the overall runtime. In addition, in order to implement optical processing, the two extra steps are rearrangement of data order and post processing (without the balanced photodetection as described in Supplementary Figure 9). The rearrangement of data order takes 7.5×10^{-6} s and the post processing takes 3.6×10^{-6} s. The sum of extra time required is 1.1×10^{-5} s, which is 0.2% of the overall runtime. Error bar of each process is defined as the standard deviation of one hundred runtimes performed in the software using MATLAB R2021b Deep Learning toolbox.

In this proof-of-concept demonstration, the photonic processor as a 2×2 preprocessor for a simple CNN is not dominating in the computational load. Yet, in some popular CNNs such as GoogleNet, AlexNet, Overheat, and VGG, the many convolution layers can take up to 90% of the overall runtime.[30] In principle, photonic processor can contribute to all convolutional processing steps to take care of computational load significantly from a futuristic perspective.

Supplementary Table 4 Comparison of experimentally demonstrated inferencing accuracies of the start-of-the-art PNNs.

Platform	Prediction accuracy		Ref
	MNIST digit	MNIST fashion product	
Integrated universal unitary network	90.5%	84.2%	28
Integrated diffractive network	91.4%	81.7%	29
Free-space diffractive network	91.75%	81.13%	31
Integrated convolutional network	86%	87%	This work

Supplementary Table 4 compares inferencing accuracies of our integrated convolutional network based on the in-memory photonic–electronic hybrid platform with those of the start-of-the-art PNNs previously reported,[28,29,31] and our network exhibits one of the highest prediction accuracies for the MNIST fashion product.

Supplementary Note 16: Scaling the dot-product engine to a photonic tensor core and the projected compute density and compute efficiency

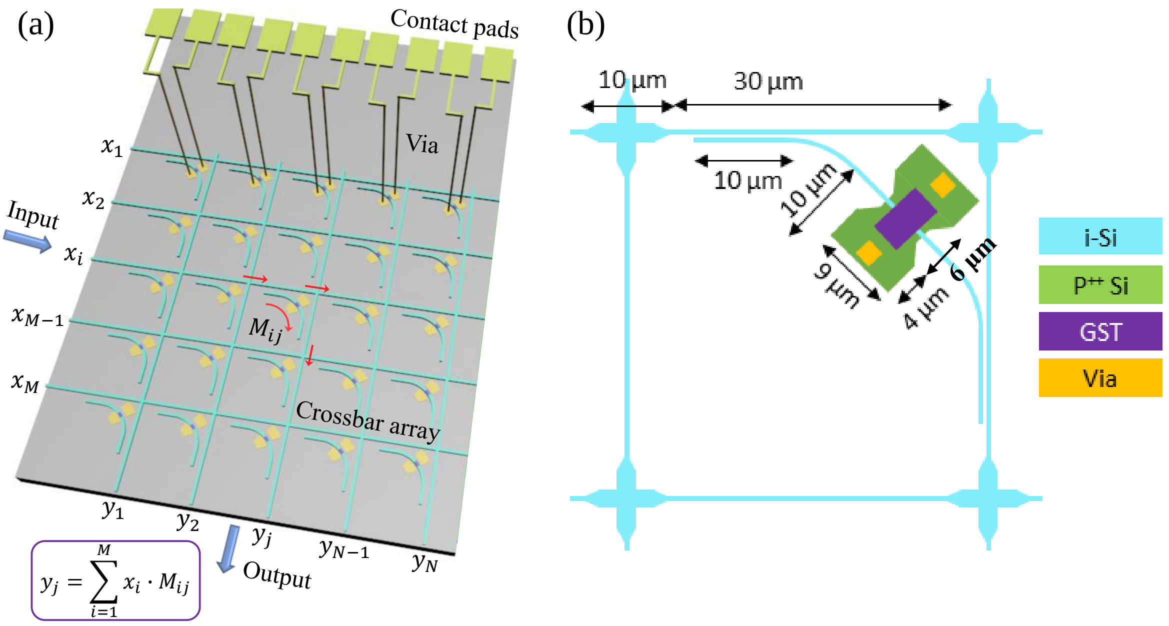

Supplementary Figure 23 The proposed photonic tensor core architecture by scaling up the dot-product engine using optical interconnects. (a) Schematic of a photonic tensor core based on the photonic–electronic hybrid integration. **(b)** A detailed unit cell with an estimated footprint less than 40 × 40 µm^2.

Supplementary Figure 23 shows the proposed photonic tensor core architecture by scaling up the dot-product engine using optical interconnects. Output (y_j) can be represented by $y_j = \sum_{i=1}^{M} x_i \cdot M_{ij}$, where M_{ij} is a matrix element encoded on the transmission of a PCM and x_i is the amplitude of an input probe signal. Thus, [y_1, y_2, … y_N] is an output vector for matrix-vector multiplication. Utilizing the parallel computing, multiple sets of input vectors can be processed in a single clock to implement matrix-matrix multiplication. Especially, this photonic–electronic platform can be controlled using microcontroller and peripheral circuits. Based on the CMOS technology, vias can be used to bridge the doped silicon device layer and upper contact metal layer for wire bonding. In this scenario, footprint of a unit cell was estimated to be less than 40 × 40 µm^2 by considering component footprints: doping region of less than 9 × 14 µm^2, bending radius of 10 µm, waveguide coupler length of 10 µm,[32] and waveguide crossing of 10 × 10 µm^2.[33] The doped waveguide is shallowly etched and other components (waveguide couplers, waveguide crossings, waveguide bends) are all fully etched, with waveguide transitions in between.

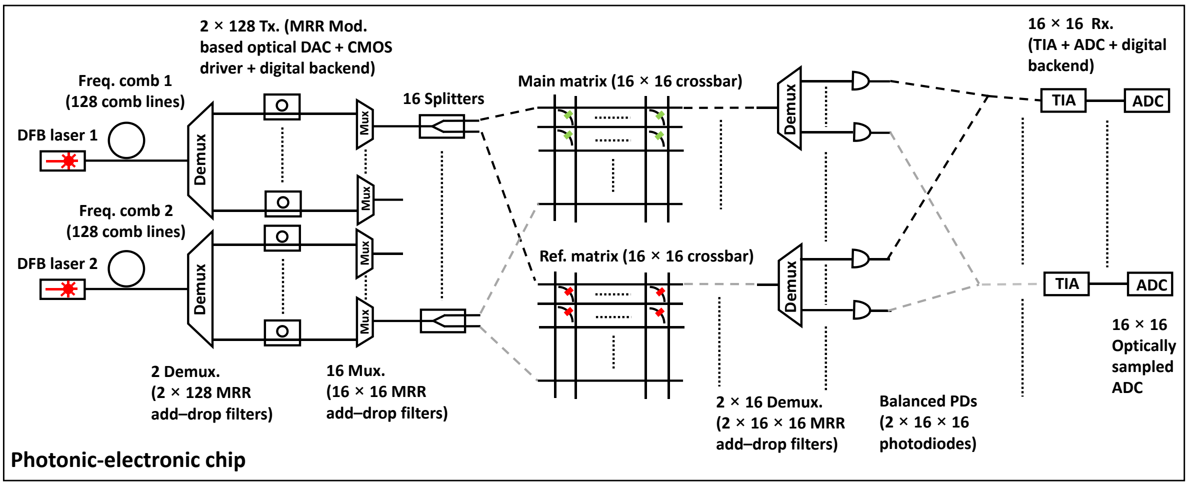

Supplementary Figure 24 A schematic showing the proposed entire photonic-electronic chip for the matrix-vector multiplication. Freq.: frequency, Tx: transmitter, Mod.: modulator, Mux.: multiplexer, Demux.: demultiplexer, MRR: microring, Ref.: reference, Rx: receiver, DFB: distributed feedback Bragg, DAC: digital-to-analog converter, PD: photodiode, TIA: transimpedance amplifier, ADC: analog-to-digital converter.

We propose a photonic-electronic chip with medium sized 16 × 16 waveguide crossbar arrays for the matrix-vector multiplication as shown in Supplementary Figure 24. And we provide reasonable estimations on compute density and efficiencies by considering sources, multiplexers, demultiplexers, transmitters (MRR-modulator-based optical DACs, CMOS drivers, thermal tuners, and digital backend), splitters, PCM crossbar arrays, receivers (photodetectors, TIAs, buffers, optically sampled ADCs, and digital backend) monolithically integrated on a silicon chip. We considered using two crossbar arrays (one main matrix and one reference matrix) and balanced photodetection scheme for non-zero-point offset correction due to the non-zero transmission at the lowest level encoded on the reference matrix. The proposed entire photonic-electronic chip as shown in Supplementary Figure 24 has digital backends, providing interfaces to an external digital electronic chip including microcontrollers and digital memory.

Supplementary Table 5 Estimated footprints and power consumptions of components in the proposed photonic-electronic chip.

Components	Area (mm^2)	Power (W)	References
DFB-frequency combs	3.92	0.7×2 = 1.4	34
Mux. and Demux. based on the FSR-free MRRs add-drop filters	2	0	35
Tx (MRR-modulator-based optical DACs, CMOS drivers, thermal tuners, and digital backend)	15.36	0.685 pJ/b × 25 × 10^9 b/s × 16 ×16 = 4.384	36
Splitters	1.28×10^{-4}	0	37
Crossbars	0.82	0	This work
Photodiodes	0.9	16×16×(0.0324+0.0165)=12.5	38
Rx.: TIAs, buffers	2.304		21
Rx.: Optically sampled ADCs, digital backend	30.72	16×16×88 (mW) = 22.528	22
Total area/power	56.02	40.81	

Supplementary Table 5 shows estimated footprints and power consumptions of components in the proposed photonic-electronic chip. Assuming a data rate of 25 Gb/s and 16 WDM

wavelength channels in parallel, estimations on compute density, compute efficiency, and energy efficiency are shown below:

Compute density:

$2\times16\times16\times(2\times16\times25\times10^9)/56.02/10^{12} = 7.3$ TOPS/mm^2

Compute efficiency:

$2\times16\times16\times(2\times16\times25\times10^9)/10^{12}/40.81 = 10.0$ TOPS/W

Energy efficiency:

$40.81/[2\times16\times16\times(16\times25\times10^9)] = 0.2$ pJ/MAC

(Total power consumed in 1 second divided by number of optical MAC operations performed in 1 second)

More details in evaluating area and energy consumption for each component are shown in the following part:

(1) DFB-frequency combs:
In our system, two laser soliton microcombs maybe needed for generating 16×16 wavelength channels (each has at least 128 comb lines), an area estimation would be taking 1/12 of the chip area for 24 integrated laser soliton microcombs[34]:
$9.8\times4.8/12 = 3.92$ mm^2
Wavelength ranges of dual combs are respectively 1520–1550 nm and 1550–1580 nm. If the comb line separation is 25 GHz, dual combs support 300 comb lines:
$(3\times10^8/1520\times10^{-9} - 3\times10^8/1580\times10^{-9})/(25\times10^9) = 299.8001$
In ref. 39, the DFB pump laser for frequency comb generation has a wall-plug electrical power of 2V × 350 mA = 0.7 W, and 2 × 0.7 = 1.4 W for two DFB-comb sources.

(2) Mux. and Demux. based on the FSR-free MRRs add-drop filters:
Four groups of 16 by 16 passive MRRs[35] are needed, they occupy an area of 1963 (μm^2) × 16 × 16 × 4 = 2 mm^2. There is no energy consumption for these passive MRRs.

(3) Transmitters:
In ref. 36, area for one transmitter (including MRR-modulator-based optical DAC, CMOS driver, thermal tuner, and digital backend) is 0.06 mm^2. Thus, total area for 256 transmitters is 16 × 16 × 0.06 = 15.36 mm^2. Energy per bit is E_{mod} = 0.685 pJ/bit. And energy consumption is estimated as: 0.685 (pJ/b) × 25 × 10^9 (b/s) × 16 × 16 = 4.384 W.

(4) Power splitters:

16 3-dB power splitters are needed to feed 50% of each input to a main crossbar and the rest to a reference crossbar. Footprint of each splitter is 2×4 μm^2.[37] And 16 splitters take an area of 16×8 μm^2 = 1.28×10^{-4} mm^2.

(5) Photonic crossbars:
Footprint of a unit cell is 40 × 40 μm^2. Then, footprint of a medium sized 16 × 16 photonic matrix is 40 × 40 × 256 = 409600 μm^2 = 0.41 mm^2. A main crossbar and a reference crossbar take an area of 0.82 mm^2.

(6) Balanced photodiodes:
It takes an area of 240 × 220 μm^2 for 5 × 6 photodiodes.[38] Thus, 2×16×16 photodiodes may take an area of 0.9 mm^2.

(7) TIAs and buffers (connecting to balanced photodiodes):
Footprint of an optical receiver Rx. (including TIA and buffer) is 0.009 mm^2. For 256 receivers: 16 × 16 × 0.009 = 2.304 mm^2. Energy consumption for each receiver is (32.4+16.5) mW, in which a buffer consumes 16.5 mW.[21] For 256 receivers, energy consumption is 16 × 16 × (0.0324 + 0.0165) = 12.5 W.

(8) ADCs:
According to ref. 22, estimated area is 0.12 (mm^2) × 256 = 30.72 mm^2. And power consumption is 256 × 88 (mW) = 22528 (mW) = 22.528 W.

Supplementary References

1 Cheng, Z. *et al.* Device-level photonic memories and logic applications using phase-change materials. *Adv. Mater.* **30**, 1802435 (2018).

2 Xiong, F., Liao, A. D., Estrada, D. & Pop, E. Low-power switching of phase-change materials with carbon nanotube electrodes. *Science* **332**, 568–570 (2011).

3 Zhang, H. *et al.* Nonvolatile waveguide transmission tuning with electrically-driven ultra-small GST phase-change material. *Sci. Bull.* **64**, 782–789 (2019).

4 Williams, J. S. Ion implantation of semiconductors. *Mater. Sci. Eng. A* **253**, 8–15 (1998).

5 Current, M. I. Ion implantation of advanced silicon devices: Past, present and future. *Mater. Sci. Semicond. Process.* **62**, 13–22 (2017).

6 Beadle, W. E., Tsai, J. C. C. & Plummer, R. D. *Quick reference manual for silicon integrated circuit technology*. (Wiley, 1985).

7 Selberherr, S. *Analysis and simulation of semiconductor devices*. (Springer, 1984).

8 Masetti, G., Severi, M. & Solmi, S. Modeling of carrier mobility against carrier concentration in arsenic-, phosphorus-, and boron-doped silicon. *IEEE Trans. Electron. Devices* **30**, 764–769 (1983).

9 Zhang, H. *et al.* Miniature multilevel optical memristive switch using phase change material. *ACS Photonics* **6**, 2205–2212 (2019).

10 Chen, R. *et al.* Broadband nonvolatile electrically controlled programmable units in silicon photonics. *ACS Photonics* **9**, 2142–2150 (2022).

11 Ding, K. *et al.* Phase-change heterostructure enables ultralow noise and drift for memory operation. *Science* **366**, 210–215 (2019).
12 Wang, X., Wu, Y., Zhou, Y., Deringer, V. L. & Zhang, W. Bonding nature and optical contrast of TiTe2/Sb2Te3 phase-change heterostructure. *Mater. Sci. Semicond. Process.* **135**, 106080 (2021).
13 Aggarwal, S. *et al.* Antimony as a programmable element in integrated nanophotonics. *Nano Lett.* **22**, 3532–3538 (2022).
14 Zheng, J. *et al.* Nonvolatile electrically reconfigurable integrated photonic switch enabled by a silicon PIN diode heater. *Adv. Mater.* **32**, 2001218 (2020).
15 Fang, Z. *et al.* Ultra-low-energy programmable non-volatile silicon photonics based on phase-change materials with graphene heaters. *Nat. Nanotechnol.* **17**, 842–848 (2022).
16 Farmakidis, N. *et al.* Electronically reconfigurable photonic switches incorporating plasmonic structures and phase change materials. *Adv. Sci.* **9**, 2200383 (2022).
17 Di Wu *et al.* Resonant multilevel optical switching with phase change material GST. *Nanophotonics* **11**, 3437–3446 (2022).
18 Salehi, S. & DeMara, R. F. Energy and area analysis of a floating-point unit in 15nm CMOS process technology. In *SoutheastCon 2015* (Fort Lauderdale, FL, USA, 2015). 10.1109/SECON.2015.7132972
19 Huang, C. *et al.* A silicon photonic–electronic neural network for fibre nonlinearity compensation. *Nat. Electron.* **4**, 837–844 (2021).
20 Tait, A. N. *et al.* Silicon photonic modulator neuron. *Phys. Rev. Applied* **11**, 064043 (2019).
21 Szilagyi, L. *et al.* A 53-Gbit/s optical receiver frontend with 0.65 pJ/bit in 28-nm bulk-CMOS. *IEEE J. Solid-State Circuits* **54**, 845–855 (2019).
22 Mehta, N. *et al.* An optically sampled ADC in 3D integrated silicon-photonics/65nm CMOS. In *2020 IEEE Symposium on VLSI Technology* (Honolulu, HI, USA, 2020). 10.1109/VLSITechnology18217.2020.9265101
23 Nayak, D., Acharya, D. P. & Mahapatra, K. Current starving the SRAM cell: a strategy to improve cell stability and power. *Circuits Syst Signal Process* **36**, 3047–3070 (2017).
24 https://www.keysight.com/us/en/assets/7018-02486/data-sheets/5990-5512.pdf.
25 Li, X. *et al.* Experimental investigation of silicon and silicon nitride platforms for phase-change photonic in-memory computing. *Optica* **7**, 218–225 (2020).
26 Feldmann, J., Youngblood, N., Wright, C. D., Bhaskaran, H. & Pernice, W. H. P. All-optical spiking neurosynaptic networks with self-learning capabilities. *Nature* **569**, 208–214 (2019).
27 Shen, Y. *et al.* Deep learning with coherent nanophotonic circuits. *Nat. Photonics* **11**, 441–446 (2017).
28 Zhang, H. *et al.* An optical neural chip for implementing complex-valued neural network. *Nat. Commun.* **12**, 457 (2021).
29 Zhu, H. H. *et al.* Space-efficient optical computing with an integrated chip diffractive neural network. *Nat. Commun.* **13**, 1044 (2022).
30 Li, X., Zhang, G., Huang, H. H., Wang, Z. & Zheng, W. Performance analysis of GPU-based convolutional neural networks. In *2016 45th International Conference on Parallel Processing (ICPP)* (Philadelphia, PA, USA, 2016). 10.1109/ICPP.2016.15
31 Lin, X. *et al.* All-optical machine learning using diffractive deep neural networks. *Science* **361**, 1004–1008 (2018).
32 Lu, L., Wu, J., Wang, T. & Su, Y. Compact all-optical differential-equation solver based on silicon microring resonator. *Front. Optoelectron.* **5**, 99–106 (2012).
33 Ma, Y. *et al.* Ultralow loss single layer submicron silicon waveguide crossing for SOI optical interconnect. *Opt. Express* **21**, 29374–29382 (2013).
34 Xiang, C. *et al.* Laser soliton microcombs heterogeneously integrated on silicon. *Science* **373**, 99–103 (2021).
35 Eid, N. *et al.* FSR-free silicon-on-insulator microring resonator based filter with bent contra-directional couplers. *Opt. Express* **24**, 29009–29021 (2016).
36 Moazeni, S. *et al.* A 40-Gb/s PAM-4 transmitter based on a ring-resonator optical DAC in 45-nm SOI CMOS. *IEEE J. Solid-State Circuits* **52**, 3503–3516 (2017).

37 Deng, Q., Liu, L., Li, X. & Zhou, Z. Arbitrary-ratio 1 × 2 power splitter based on asymmetric multimode interference. *Opt. Lett.* **39**, 5590–5593 (2014).

38 Ashtiani, F., Geers, A. J. & Aflatouni, F. An on-chip photonic deep neural network for image classification. *Nature* **606**, 501–506 (2022).

39 Xiang, C., Jin W., Guo J., Peters J. D., Kennedy M. J., Selvidge J., Morton P. A., & Bowers J. E. Narrow-linewidth III-V/Si/Si_3N_4 laser using multilayer heterogeneous integration. *Optica* **7**, 20–21 (2020).
